# Collaborative networks in community-based health and social care services: insights from Blackpool and the Fylde Coast (United Kingdom)

**DOI:** 10.1186/s12961-025-01303-1

**Published:** 2025-03-17

**Authors:** Kristof Santa, Zsofia Boda, Buket Kara, Jörg Huber, Heather Catt, Barbara Mezes

**Affiliations:** 1 Department of Primary Care and Mental Health, Institute of Population Health, University of Liverpool, 74 Bedford Street South, Liverpool, L69 7ZA United Kingdom; 2 Department of Sociology and Criminology, University of Essex, Wivenhoe Park, Colchester, CO4 3SQ United Kingdom; 3 Division of Health Research, Lancaster University, Health Innovation One, Sir John Fisher Drive, Lancaster, LA1 4AT United Kingdom; 4 School of Education, Sport and Health Sciences, University of Brighton, Falmer, Brighton, BN1 9PH United Kingdom; 5 Blackpool Teaching Hospitals (NHS Foundation Trust), Trust Headquarters, Whinney Heys Road, Blackpool, FY3 8NR United Kingdom

**Keywords:** Community-based services, Health care, Social care, Collaboration, Social network analysis, England, Integrated care, Health equity

## Abstract

**Background:**

Globally, health and social care systems have been responding to the demand for better integrated service delivery to tackle complex public health and socioeconomic challenges. Similarly, services in the United Kingdom strive for comprehensive, person-centred care to support health equity and improved quality of life. This study took place in Blackpool and the Fylde Coast, United Kingdom, where socioeconomic deprivation and health inequalities persist and effective collaboration among health and social care providers offers an opportunity to tackle such complex challenges. The study used social network analysis (SNA) to investigate collaboration patterns between organizations to identify key characteristics and areas for improved integration.

**Methods:**

Data were collected from March to June 2023. First, a comprehensive mapping exercise identified a total of 453 community-based providers who were invited to participate via email. Data on service provision were collected using an adapted version of the Template for Intervention Description and Replication (TIDieR) form from organizations’ websites. Service descriptions were thematically categorized into 11 domains. A total of 44 organizations provided information on their collaborations through an online survey, reporting on collaborations across 321 organizations. SNA examined collaboration patterns via visualization and multivariate network regressions (MRQAP).

**Results:**

The mapping identified a great range of community-based support. The network density indicated relatively low overall collaboration (2.2%) among 321 organizations. Within the subset of 44 organizations who completed the questionnaire, collaborations were more frequent (15%). Collaboration ties were unevenly distributed, where some organizations had more connections. MRQAP showed that organizations within the same domain were more likely to collaborate. Some combinations, such as collaborations between housing, shelter and nutritional support with child and family support and mental health were significantly overrepresented.

**Discussion:**

The network had low density, highlighting the potential for more collaborations. The network appears fragmented, probably owing to a tendency for organizations to collaborate with others operating in the same service domain. The frequent collaborations between certain domains highlight the complex needs of local communities. Effective integrated care initiatives, data sharing and place-based partnership/voluntary, community, faith, social enterprise sector capacity-building programmes could build more resilient and interconnected networks that meet community needs.

## Background

Integrating health and social care service planning and delivery to meet increasingly complex and diverse care needs is a global public health priority [1]. Research in this area suggests that integration may enhance perceived quality of care, increase patient satisfaction and improve access to care and health outcomes [2]. The National Health Service (NHS) Long-Term Plan (2019) aimed for more integrated health (primary care, secondary care, specialist care and mental and physical health) and social care services, encouraging shared responsibility across systems to enhance population health outcomes [3]. This marked a significant policy shift, moving from a competitive framework to one of collaboration.

The Health and Care Act (2022) built upon these foundations and created integrated care systems (ICSs) [4]. ICSs are statutory partnerships of health and care organizations. They aim to plan, design and deliver more comprehensive care; improve outcomes in population health and care; tackle inequalities in outcomes, experience and access; enhance productivity and value for money and to help the NHS to support broader social and economic development [5]. ICSs are made up of integrated care boards (ICBs) and integrated care partnerships (ICPs) [5]. ICBs are the NHS organizations responsible for planning healthcare services [6]. ICPs are joint committees between NHS organizations and local authorities in each ICS, which includes the voluntary and other sectors with a role in improving local health, care and wellbeing [6]. ICPs develop long term strategies to collectively improve services, health and wellbeing and reduce inequalities within geographical regions [6]. Although not legislated, place-based partnerships (PBPs) operate in larger ICSs involving the NHS, local government and the voluntary, community, faith, social enterprise (VCFSE) sector with an aim to design and deliver integrated services that meet the distinctive needs of local populations within communities [5].

Community-based health and social care services in the United Kingdom can meet the NHS Long-Term Plan’s objectives to deliver personalized services outside of hospital settings to promote the independence of populations whilst reducing the demands on acute health services [7, 8]. They support vulnerable groups including the elderly [9], low-income families [10] and individuals with chronic conditions [11]. Whilst health and care services are important, it is widely recognized that most of the factors which determine the health of the population lay outside of healthcare [12].

Community-based support is often delivered by the VCFSE sector. The VCFSE has extensive neighbourhood knowledge; they are close to communities, have skills in and experience of working with the most disadvantaged people, including underserved communities, and have the flexibility to respond to community needs and deliver effective interventions at a lower cost than other sectors [13]. VCFSE services have the potential to ease the burden on health and social care services, and health and care services frequently leverage available resources and partnerships with the VCFSE to address the wider determinants of health, such as housing and employment [13, 14].

The current study focuses on the Fylde Coast, in the Northwest of England, which was the footprint of the local PBP until July 2022. After this time, the Lancashire and South Cumbria ICB leadership changed the footprint of the PBP to cover Blackpool. By doing so, the footprint of the PBP aligns with the footprint of the health and wellbeing board, which is a further mechanism for local strategic partnership working since introduced for all local authorities with social care and public health responsibilities under the Health and Social Care Act of 2012.

The Fylde Coast, particularly Blackpool, has notable pockets of deprivation which drive poor population health, along with its significantly ageing population [15]; 24.6% of Fylde Coast residents are aged 65 years and above, higher than the England average of 18.5% [16]. The population has limited ethnic diversity, with only approximately 5% of the population having an ethnic minority background [17]. The Fylde Coast faces many challenges including high unemployment rates and low income in employment owing to the preponderance of seasonal and part-time employment opportunities [18, 19], high rates of homelessness and poor-quality private sector housing [20] and poor health outcomes [15]. Statistics indicate conditions worse than the national average, including life expectancy, mental health outcomes [21] and education and employment indicators, such as school attainment, exclusion rates and unemployment rates [22].

Blackpool and the Fylde Coast have a strong VCFSE sector, but it does not have any sort of infrastructure organization to coordinate and foster collaboration and capacity-building [23]. By pooling resources, organizations can offer a broader range of services without duplicating efforts [24], whilst improving health outcomes [25] and enhancing the efficiency and comprehensiveness of care, particularly in resource-constrained environments [26]. Successful collaboration on the Fylde Coast occurred during the coronavirus disease 2019 (COVID-19) pandemic [27], and there are multiple ongoing initiatives in Blackpool that seek to improve the wider determinants of health and population health.^1^

Such initiatives demonstrate how well-structured networks can have important roles in aligning diverse efforts of various organizations [28]. However, there is a gap in systematically exploring and understanding the interplay of organizations and services that provide community-based support. The contributions of community-based organizations play a critical role in the Fylde Coast’s health and social care ecosystem [29], but are often underrepresented in literature [30]. This creates a structural, cultural and value-based divide with a significant impact on the availability of non-healthcare research [31–33]. These gaps in the literature conceptualize broader issues that include the limited investigations into the trends and patterns of collaborations among community-based health and social care organizations in local partnerships. Previous evaluations and studies offered only partial insights into how collaborations occurred in practice, with studies preferencing impacts and outcomes of collaborations rather than the methods and dynamics that contributed to these successes [34]. Understanding such patterns is crucial for recognizing relevant practices and barriers to beneficial collaborations. Exploring organizational interactions may aid, for example, in identifying favourable approaches to information sharing [35], subsequently contributing to the coordination of care, whilst gaining valuable insights into the effectiveness of integrated care models [36].

Social network analysis (SNA) is the ideal methodology to explore patterns of interactions between organizations within a defined setting [37]. It has several benefits; it is an objective, replicable representation of the community, and it provides a systematic understanding of local networks and the relationship of local organizations [38]. SNA provides a framework to explore relationships of social structures in a network [38]. It provides information on network characteristics, including size (number of organizations and connections), cohesiveness (number of distinct groups) and centrality (position of different organizations in the network) enabling the examination of networks of collaborations within and between community-based health and care services [39].

Previous research demonstrated the effectiveness of SNA in identifying strategies to improve collaborations between organizations in health and social care contexts [39–43]. SNA was also found to be useful in revealing strong, weak and unacknowledged ties and to examine how information and resources are distributed in a network. SNA provides network illustrations, which can pinpoint where the networks could be strengthened and establish more effective strategies to enhance service delivery [44]. For example, researchers could explore how a comprehensive network structure can contain multiple actors that are positioned centrally and on the periphery in the network, suggesting the importance of maintaining the effectiveness, resilience and vitality of the entire network rather than sole actors only [45].

## The current study

This study aimed to map the range of community-based health and social care service providers on the Fylde Coast to examine the collaborations across service domains and organizations. The objectives were to: (i) better understand the range and type of health and social care services that are currently being delivered in the local area; (ii) explore and analyse the collaboration patterns of organizations and service domains within the PBP and (iii) provide methodological considerations for using SNA to examine the nature and level of integration of health and social care networks.

## Methods

### Design

A comprehensive mapping exercise and SNA were undertaken.

### Data collection

The methods of the comprehensive mapping exercise were adapted from peer-reviewed research [46], and the exercise was conducted between March and May 2023. Service providers were identified through expert knowledge provided by project team members and using search terms on Google, Google Maps, Facebook and Twitter. The specific search terms used are provided in Supplementary Material 1. Additionally, hand-searches were conducted on the websites of local authorities, VCFSE organizations and the NHS.

Identified services were approached via email between May and June 2023. This email contained information about the study and a link to a Qualtrics online survey, which included the participant information sheet, consent form and an adapted version of the Template for Intervention Description and Replication (TIDieR) form [47]. The adapted TIDieR form, detailed in Supplementary Material 2, included a 26-item questionnaire to collect comprehensive data on each provider’s health and social care activities. The form included a roster of service providers to indicate continuous collaborations (social network data), types of populations supported, modes of engagement and organizational participation in research and evaluation. Three reminder emails were sent if no response was received.

### Data processing

The collected social network data on collaborations were cleaned by removing duplicates, correcting inconsistent formatting, standardizing entity names and checking for missing values and errors. Service providers were contacted if they submitted incomplete or erroneous data, ensuring a final dataset without missing values. Information provided with abbreviations, acronyms and partial acronyms was standardized to avoid duplications. Each service provider was assigned a unique ID to maintain confidentiality.

### Ethical considerations

Ethical approval was granted by the Ethics Committee of the University of Liverpool. Reference no.: 12221. Each participant, whether a service provider or a representative of a service provider, provided written consent to participate in the study.

### Data analysis

Service descriptions collected via the adapted TIDieR form and additional information collected from each organization’s website during the provision mapping stage underwent content analysis to generate service domains used to categorize each service provider. Each organization was categorized into a single service domain on the basis of their most prominent feature (for example, Name Church – religious and spiritual organizations) to avoid redundancy and use clarity and consistency in reporting. These service domains facilitated the grouping of service providers within the social network. Social network analysis was utilized to examine the data on collaborations, allowing the exploration of interaction patterns among service providers [48]. This methodology provided an objective, replicable representation of the community-based service providers and a systematic overview of local networks and provider relationships [48].

The following methods of social network analysis were used: (1) visualization; (2) descriptive network analysis and (3) linear regressions for network data. The analyses focused on nodes, that is, the organizations, and ties between them, that is, the collaborations. For visualization, the igraph package was used within R, which allows for the visual representation of the network as a graph. The visualization relied on the Fruchteman–Reingold algorithm, a force-directed graph drawing algorithm that positions nodes by assigning forces among the set of edges and the set of nodes on the basis of their relative positions, in an aesthetically pleasing way (for example, ties are similar in length with as few crossing ties as possible) [49].

For descriptive statistics, the network density, as well as overall and service domain-based centralities were calculated. Density expresses the number of ties that exist in the network compared with the number of potential ties that could exist if each organization was tied to each other organization. The analysis focused on two types of centralities: degree and betweenness. Degree centrality expresses the number of organizations each organization is tied to. Betweenness centrality shows a more nuanced picture of the organization’s position in the network and is expressed by the proportion of shortest paths between each pair of other organizations that pass through the organization in question, where a shortest path between two organizations means the shortest possible chain of ties through which one organization could “reach” another. Betweenness centrality can therefore be understood as a measurement of influence or power [50]. For this, the igpaph R package was used. Finally, for network regressions, the multiple regression quadratic assignment procedure (MRQAP) framework was used, which is a regression framework specifically developed for network data [51]. Using a specialized approach is necessary because standard regression methods assume the independence of observations, whereas observations in network data – network ties – show strong dependencies with each other. The MRQAP approach deals with these dependencies by offering a permutation test to assess statistical significance [52]. This analysis was conducted using the sna R package.

## Results

### Mapping exercise

**Fig. 1:**
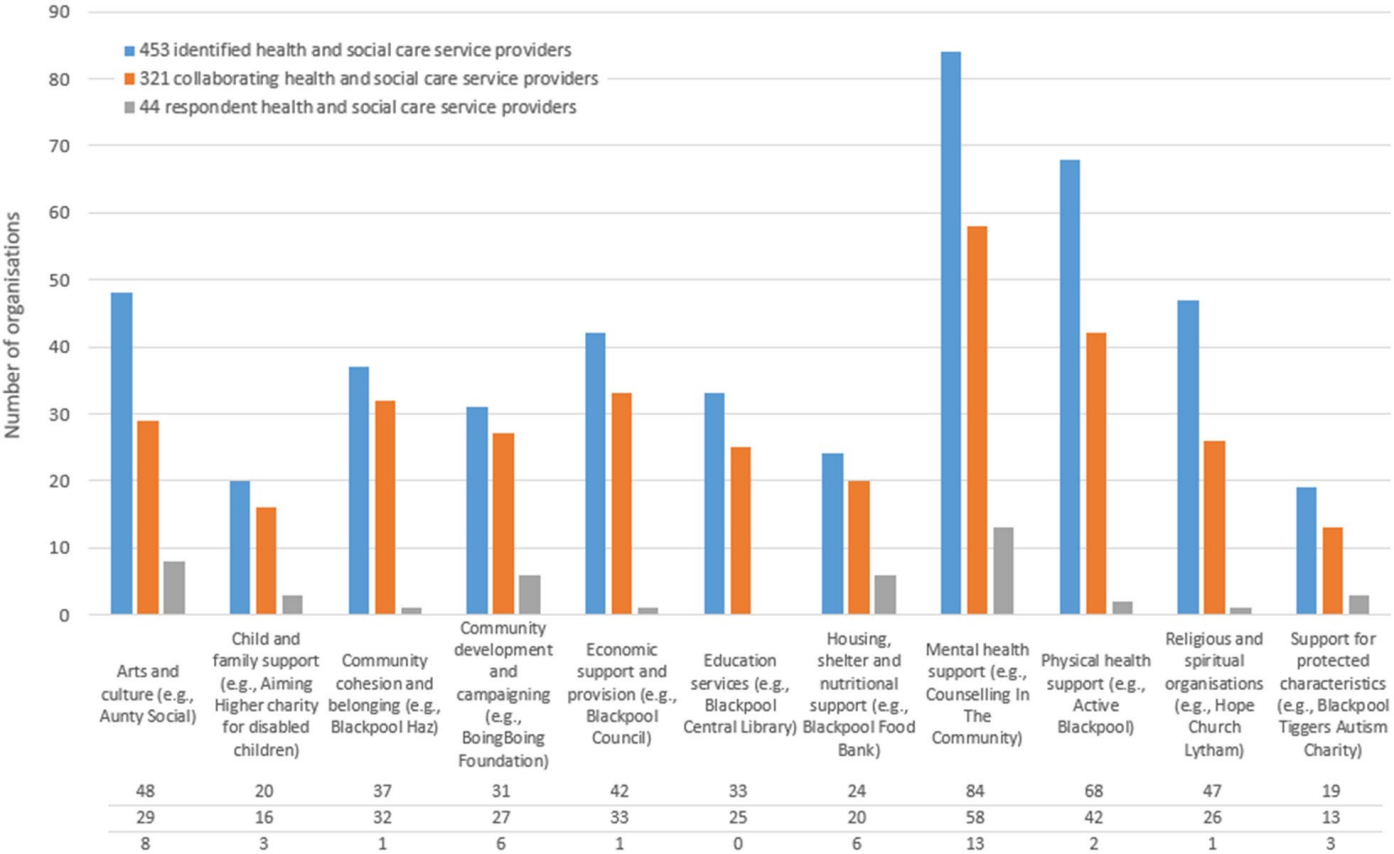
Distribution of health and social care service providers across the service domains

The mapping identified 453 organizations providing community-based health and social care support on the Fylde Coast. The organizations were thematically categorized on the basis of their primary service domain, which resulted in 11 service domain categories (Fig. 1). Out of the 453 organizations, 44 participated in the study (subset) and provided information on their collaborations across a total of 321 organizations (alters) (see Table 1).

**Table 1:** Sectoral breakdown of the participating and collaborating organizations

	VCFSE	NHS	Private sector	Public sector
Participating organizations	37	2	2	3
Collaborating organizations	221	32	17	51

### Social network analysis

#### Treatment of the network as nondirected

Collaboration ties are inherently symmetrical (if organization A collaborates with organization B, organization B collaborates with organization A). Therefore, a collaboration tie was considered to exist if reported by at least one organization. This approach allowed inclusion of collaborations involving organizations that did not participate in data collection.

However, the data was checked for reciprocity (the proportion of ties reported by both parties) in cases where both organizations completed the questionnaire. Only 27% of reported collaborations were also mentioned by the partner organization. This poses a problem, as it suggests that we observe only 27% of ties that nonparticipating organizations might have reported, assuming similar patterns. Consequently, collaborations of questionnaire respondents are overrepresented, potentially introducing bias into the dataset.

Two strategies were employed to address this issue. For visualizations and descriptive analyses, all analyses were conducted first on the full sample and then on the subset comprising only organizations that provided responses (to avoid participation bias). In regression analysis, participation (whether the organization responded to the questionnaire) was included as a variable to mitigate bias.

#### Network descriptives

**Fig. 2:**
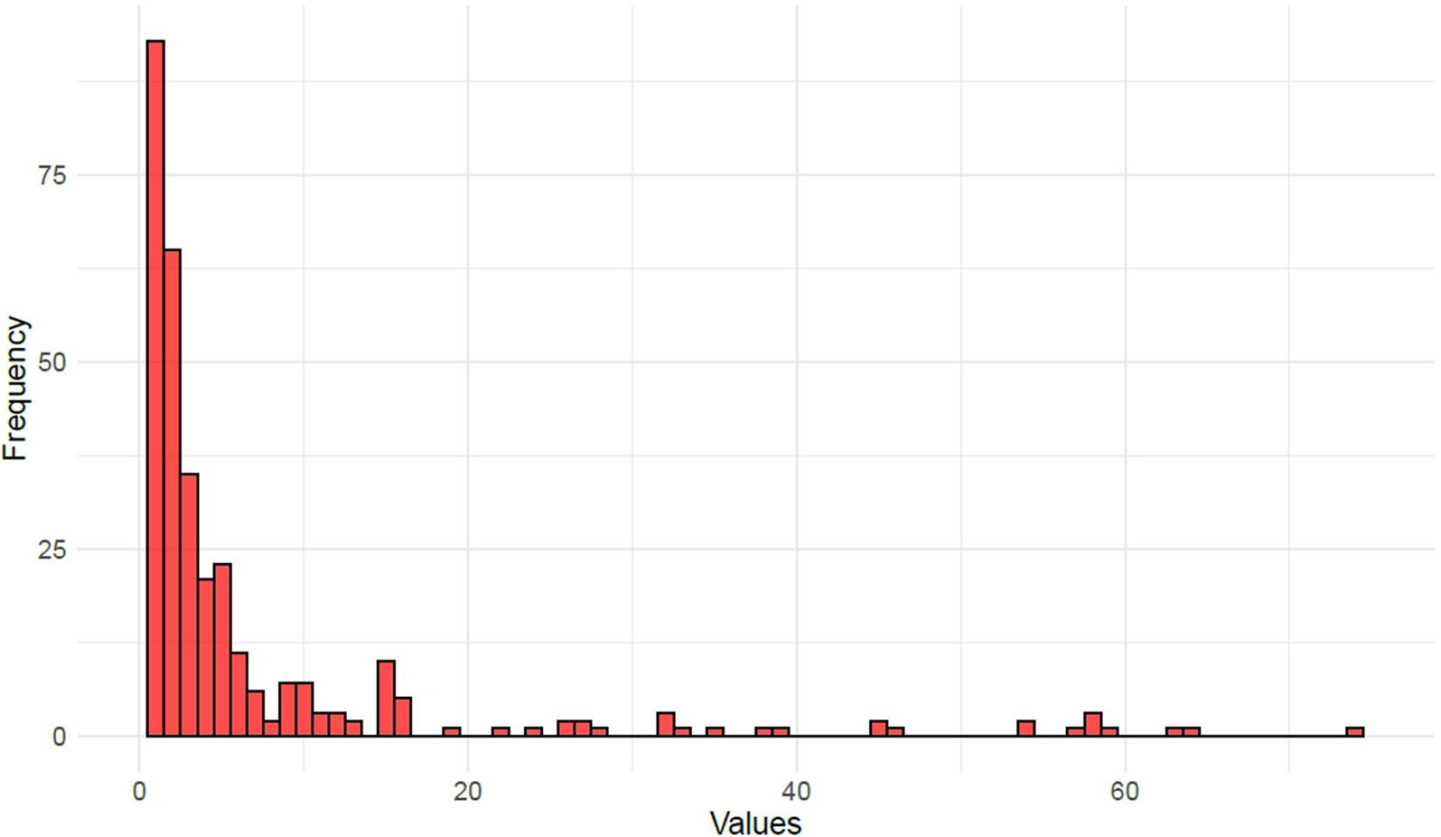
Degree distribution of organizations by frequency

The density of the network – the number of actual collaborations compared with the potential number – is 2.2%. This is a low percentage figure, but not unexpectedly, as organizations are unlikely to be able to collaborate with hundreds of others. The number of collaborations is unevenly distributed in the network, known as the degrees of individual organizations. The degree distribution is shown by Fig. 2, where every bar stands for a degree and the height of the bar shows the number of organizations with that degree. The maximum degrees per organization is 74, but the majority of organizations have low degrees – collaborations between one and five.

**Fig. 3:**
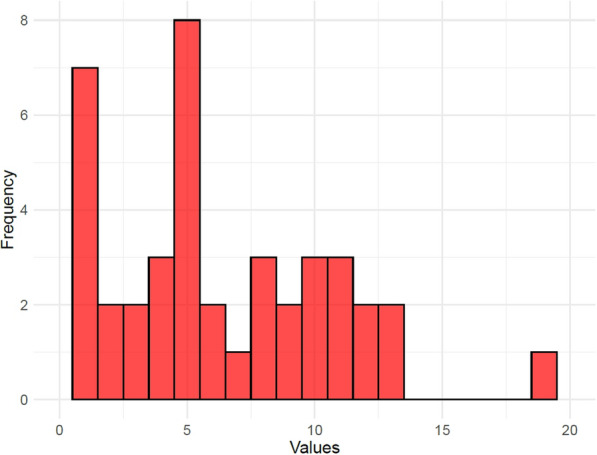
Degree distribution in respondent subset with 44 organizations

Within the subset of respondents, the density is 15%. This shows that collaborations are common within this smaller group of respondent organizations. The degree distribution is less uneven in this subset; this is shown by Fig. 3. The maximum degree among the 44 organizations is 19.

**Fig. 4:**
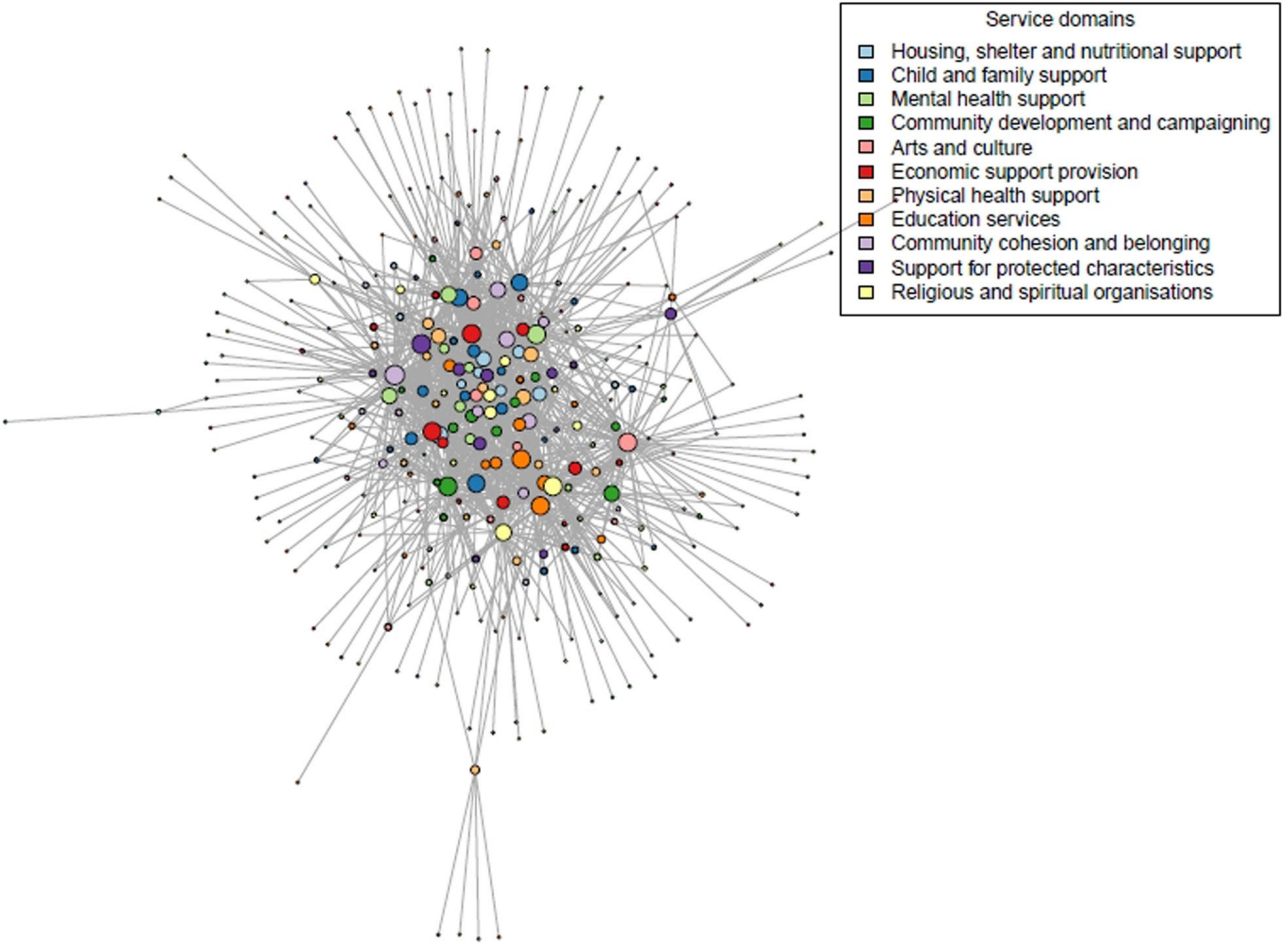
Network plot of the whole network

Network plots were created to visually assess the structure of the network. Figure 4 shows the plot for the whole network. Larger node sizes stand for an organization with more collaborations (on a logarithmic scale); colours show the service domain of the organization.

**Fig. 5:**
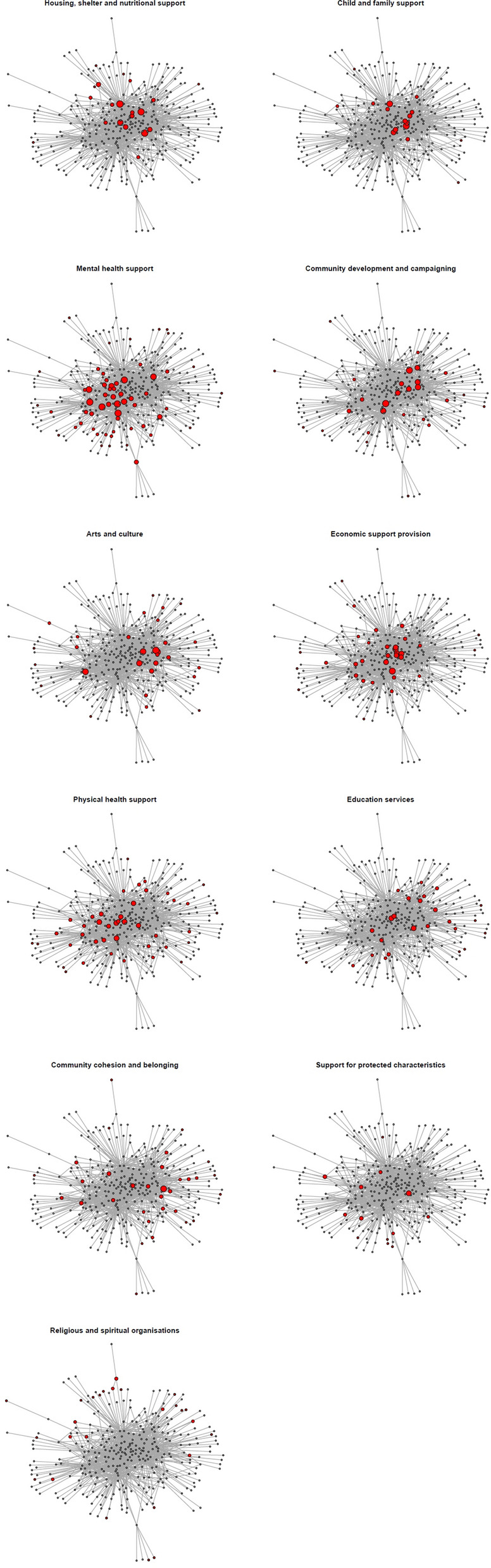
Network subsets by service domain

Figure 5 presents subsets of the networks for each service domain to enable visual assessment of the position of organizations. Red nodes show organizations within the service domain; white nodes are other organizations. Sizes for the red nodes show how many partners the organization has (sizes do not vary for the non-highlighted white nodes). The plots indicate that some types of organizations (housing, shelter and nutritional support; child and family support and mental health support) are on average more central, whereas some (especially religious and spiritual organizations) are less central than others.

**Fig. 6:**
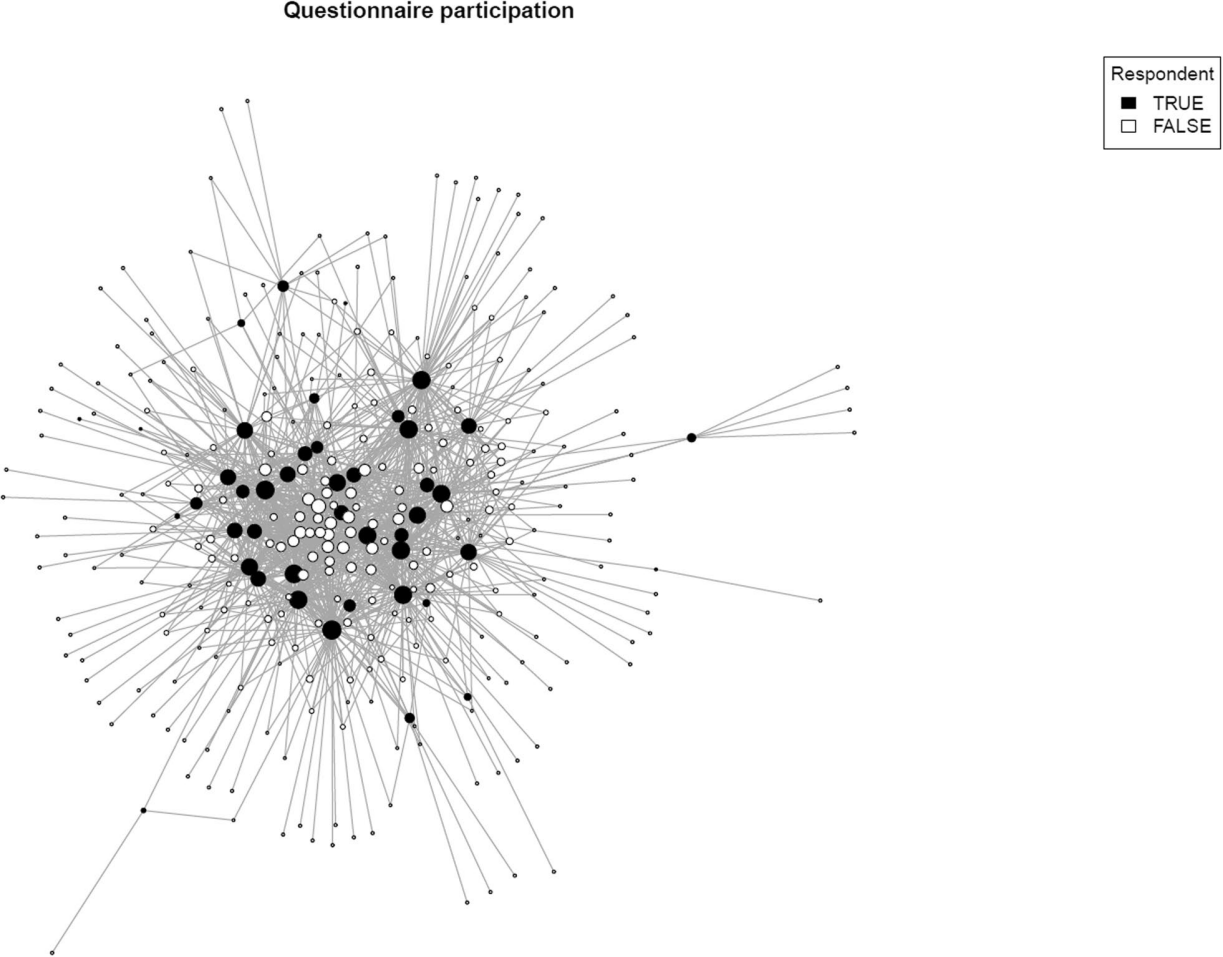
Centrality of questionnaire respondents compared with nonrespondents

However, Fig. 6 shows that, as expected, those who responded to the questionnaire (blue nodes) appear much more central than those who did not. Therefore, apparent differences between service domains may be due to differences in participation rate by sections.

**Fig. 7:**
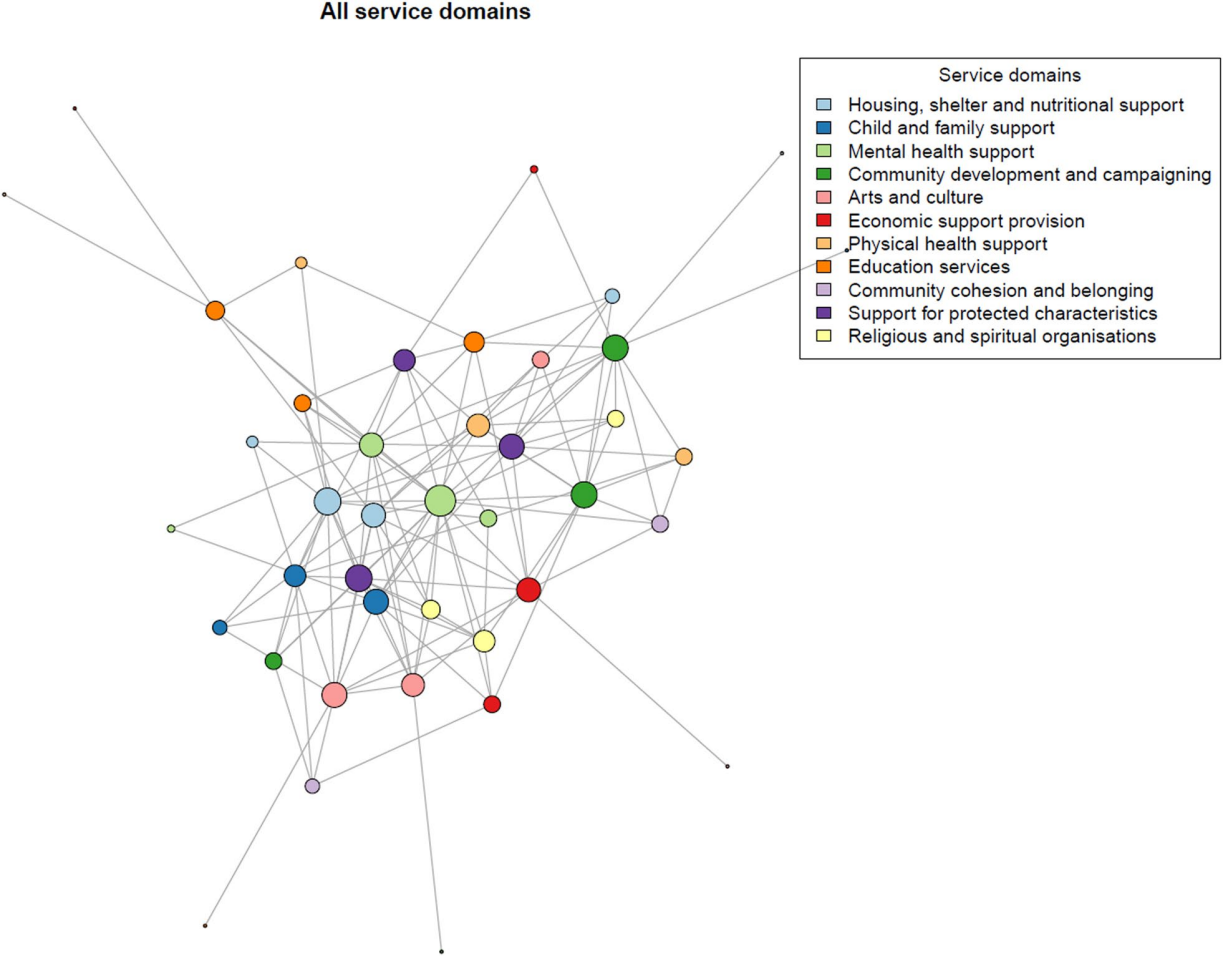
Network plot of questionnaire respondents

**Fig. 8:**
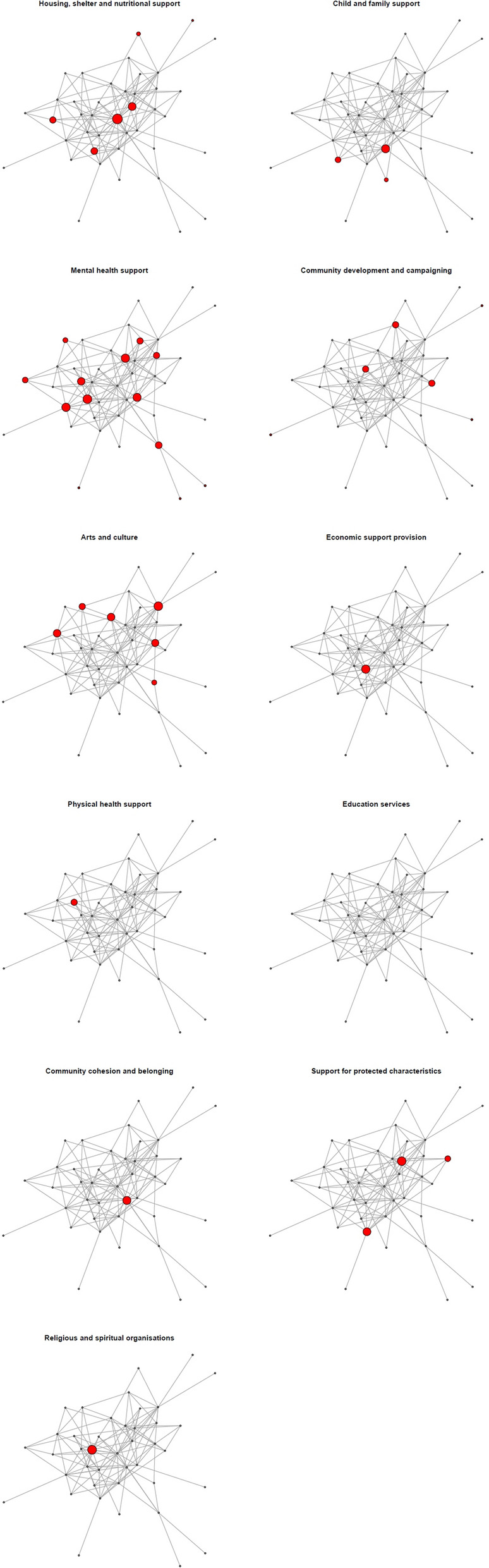
Network subsets by service domain for questionnaire respondents

Figures 7 and 8 replicate Figs. 4 and 5, respectively, for the respondent subset of the network. Here, clear between-service domain differences cannot be identified, which may be due to the smaller sample.

**Fig. 9:**
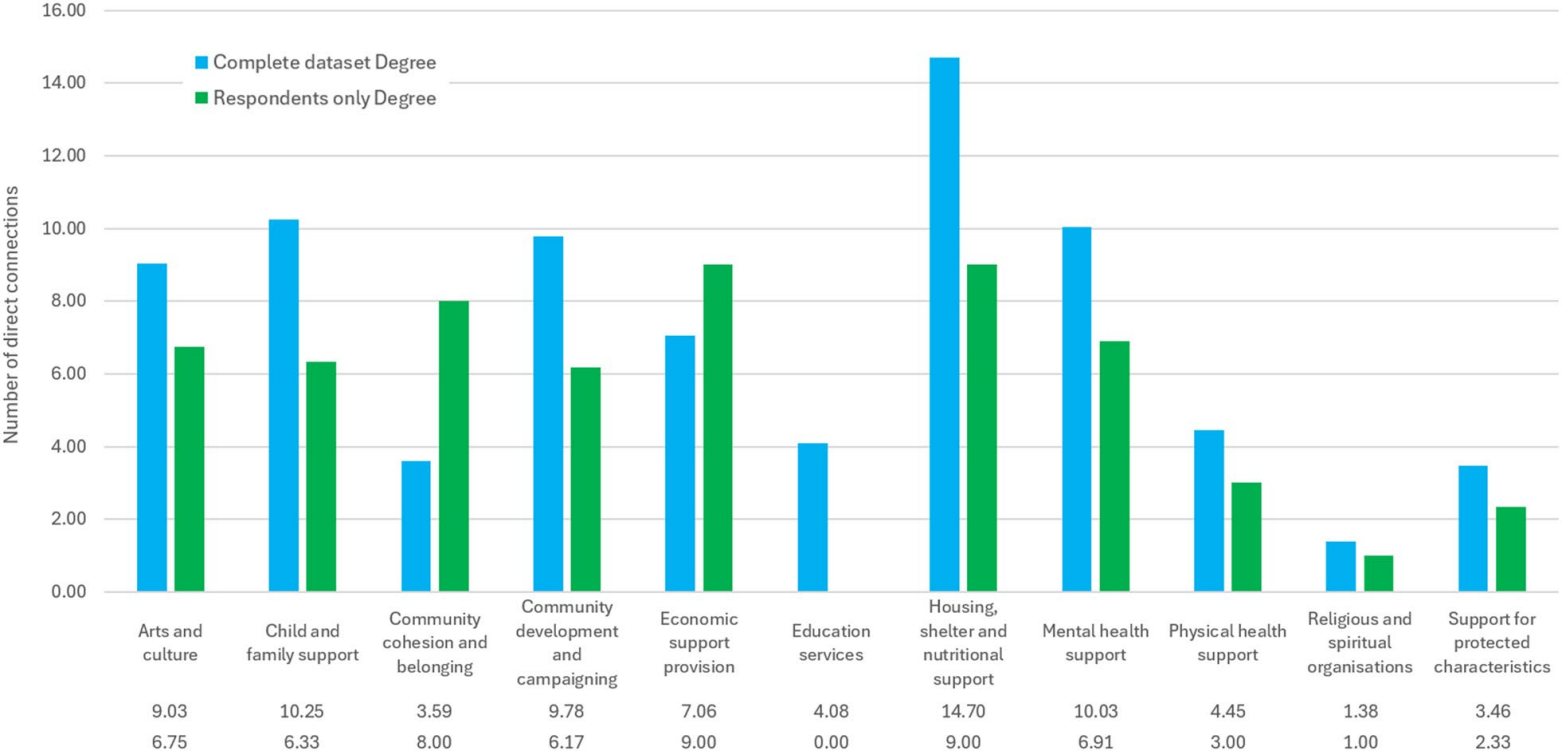
Mean degree by service domain

**Fig. 10:**
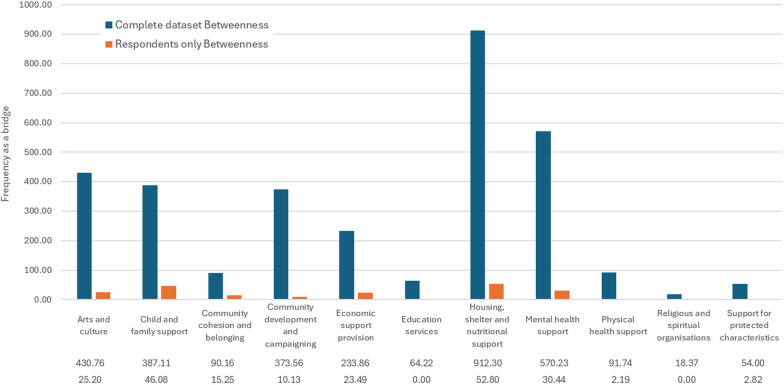
Betweenness centrality by service domain

This is also supported by descriptive service domain-wise centrality results, shown by Figs. 9 and 10, which show the mean degree and betweenness centralities of organizations. Housing, shelter and nutritional support seems to be the most central service domain in both datasets, both in terms of degree (the pure number of relationships) and betweenness (the importance of the organization as a bridge between other organizations) in both datasets. However, economic support provision appears similarly important degree-wise (but not betweenness-wise) in the respondent subset, with community cohesion and belonging organizations also being more central in the respondent subset (especially degree-wise). Apart from this, the order of service domains seems similar across centrality types and datasets.

#### Multivariate regression analysis

A total of four network regression models (MRQAP) were estimated and tested for statistical significance to assess service domain-wise differences between collaboration patterns whilst controlling for respondent status. The dependent variable in each model is collaboration ties: how likely it is for a collaboration to exist between two organizations, given the independent variables. A dummy variable was included to control for whether the organization responded to the questionnaire (value 1) or was mentioned by others (value 0).

Model A (Table 2) includes service domain main effects only, showing how likely an organization is to collaborate with any other organization given its own service domain membership. The reference category is religious and spiritual organizations; for the other categories, additional probabilities are estimated compared with this. No significant differences between service domains were found in overall collaboration activity when respondent status was included as a control variable. This was a consistent finding across all models.

**Table 2:** Model A – service domain main effects

	Model A	
	Coefficient (*β*)	*p*-Value
Intercept (reference category: religious and spiritual organizations)	0.013	0.90
Sector main effects		
Arts and culture	−0.003	0.79
Child and family support	−0.006	0.55
Community cohesion and belonging	−0.004	0.70
Community development and campaigning	0.000	0.97
Economic support provision	0.001	0.92
Housing, shelter and nutritional support	0.005	0.72
Mental health support	0.010	0.41
Physical health support	−0.020	0.10
Religious and spiritual organizations	−0.011	0.28
Support for protected characteristics	0.007	0.50
Education services	No data	No data

^*^statistically significant

Model B incorporated homophily as a variable, indicating whether service domains preferentially collaborated within their own domain. The results showed a positive and statistically significant effect (*β* = 0.015, *p* < 0.001), confirming that organizations were more likely to collaborate within than outside their domains.

Model C (Table 3) considered homophily tendencies for each service domain separately. The main homophily effect refers to the reference category (homophily within the religious and spiritual organizations domain), which is not significant, showing no overrepresentation of same-service domain ties compared with cross-service domain ties within the reference category. Statistically significant homophily was found within housing, shelter and nutritional support (*β* = 0.094, *p* < 0.001), child and family support (*β* = 0.050, *p* < 0.001), mental health support (*β* = 0.099, *p* < 0.001), community development and campaigning (*β* = 0.039, *p* = 0.02) and economic support provision (*β* = 0.043, *p* = 0.01). No significant homophily was found for arts and culture, physical health support, education services, community cohesion and belonging or support for protected characteristics – organizations within these service domains appear equally likely to collaborate within their service domains as outside of it.

**Table 3:** Model C – homophily within sectors

	Model C	
	Coefficient (*β*)	*p*-Value
Intercept (reference category: religious and spiritual organizations)	0.014	0.90
Arts and culture	0.000	0.98
Child and family support	0.050	< 0.001*
Community cohesion and belonging	0.010	0.46
Community development and campaigning	0.039	0.02*
Economic support provision	0.043	0.01*
Housing, shelter and nutritional support	0.094	< 0.001*
Mental health support	0.099	< 0.001*
Physical health support	0.003	0.88
Religious and spiritual organizations	0.012	0.38
Support for protected characteristics	0.012	0.34
Education services	No data	No data

^*^statistically significant

Finally, in Model D (Table 4), other interactions are included to look at collaborations between pairs of service domains; namely, between each of the five service domains that were most active in collaborations according to descriptive analysis results. Here we find significantly more collaborations between housing, shelter and nutritional support and child and family support (*β* = 0.019, *p* = 0.05), as well as between housing, shelter and nutritional support and mental health support (*β* = 0.015, *p* < 0.001) compared with the reference category (religious and spiritual organizations collaborating with each other). The rest of the results do not change in this model; there are no significant service domains main effects, but there are various significant service domain-specific homophily effects.

**Table 4:** Model D–collaborations within specific sectors

	Model C	
	Coefficient (*β*)	*p*-Value
Intercept (reference category: religious and spiritual organizations)	0.014	0.90
Housing, shelter and nutritional support × child and family support	0.019	0.05*
Housing, shelter and nutritional support × mental health support	0.060	< 0.001*
Housing, shelter and nutritional support × community development and campaigning	0.011	0.27
Housing, shelter and nutritional support × arts and culture	−0.001	0.91
Child and family support × mental health support	0.007	0.51
Child and family support × community development and campaigning	0.014	0.13
Child and family support × arts and culture	−0.007	0.36
Mental health support × community development and campaigning	−0.002	0.78
Mental health support × arts and culture	−0.002	0.78
Community development and campaigning × arts and culture	−0.013	0.09

*statistically significant

## Discussion

The current study explored collaborations and their patterns among community-based health and social care service providers in Blackpool and the Fylde Coast. The findings illustrated a network characterized by relatively low density and an important overrepresentation of within-domain versus cross-domain collaborations.

### Mapping local service provision on the Fylde coast

A comprehensive mapping exercise was carried out to inform the public, local service providers and policymakers on what is being delivered. This is particularly important in an area of high need to identify services available to the public, inform link workers and other referrals promising practices or gaps in provision. A total of 453 organizations and their service provision were thematically categorized into 11 service domains, suggesting a great range and diversity in community-based service provision.

### Understanding collaboration patterns

The findings revealed a 2.2% network density in interorganizational collaborations. This figure may seem low, but network density is very sensitive to the size of the network, as the number of potential ties (if each organization collaborated with all other organization) is very high, and the number of actual collaborations is rarely expected to increase proportionately as the number of available partners grows. Given the large number of organizations identified, 2.2% means that an average organization had almost 7 partners, which is not necessarily a low number but may indicate space for more collaborations to develop. However, these ties tend to cluster within service domains: we detected significant homophily whereby organizations tend to partner with organizations from the same domains rather than other domains. This was true for housing, shelter and nutritional support, child and family support, mental health support, community development and campaigning and economic support provision.

The importance of this is twofold. First, there seems to be less collaboration across domains, which contrasts with the comprehensive care proposed by legislations, as well as the ICS [53], indicating a potential discrepancy between the ideal and observed integration. Current public health challenges require more inter- and trans-disciplinarity to tackle the complex interplay between social, psychological, economic and environmental factors driving both mental and physical health outcomes, highlighting the need for improved cross-domain collaborations [54]. Second, we found more collaborations between domains which align with the priority areas of the PBP; housing, mental health, first 1001 days and education, employment and skills. This may highlight the importance of high-level buy-in and investment of resources and support for priority areas to translate into more collaborations between these domains.

The dispersion of collaborations demonstrated an uneven distribution of resources, where a few organizations had a high number of connections, whilst most of the organizations had relatively few. This disparity highlighted individual organizations that appear to be more isolated. The meaningful differences in connections may be driven by differing capacities, strategic priorities or resource availability. The key role of central organizations may suggest that the network’s overall connectivity is dependent on the sustained engagement and survival of these service domains [55]. Losing these organizations could result in a significant fragmentation of the network, resulting in partnerships ceasing to exist and consequently reducing further collaborations of other organizations who relied on these specific clusters in terms of resources and support [56]. Strengthening the underlying fabric of the network ensures the continued involvement of central organizations, whilst allowing underintegrated organizations to build capacity and take on more active roles [28]. However, in areas like Blackpool, where the VCFSE sector lacks a strong infrastructure organization such as a council for voluntary service, challenges arise in fostering collaboration, sharing resources and building capacity. This absence underscores the importance of infrastructure organizations in sustaining a connected and resilient sector [57].

Furthermore, the implementation of strategic frameworks by ICSs can inadvertently lead to the emergence of dominant clusters. These clusters may represent a barrier of effective collaboration in the form of hegemonic visions, accountability mechanisms and values [53]. In Blackpool, this dynamic is further compounded by the aforementioned infrastructure void, as organizations lack the strategic guidance and support typically offered by a coordinating body. This has left the sector reliant on ad hoc efforts rather than a sustained, integrated approach to overcoming systemic barriers. A stronger, sector-wide infrastructure is therefore essential to enable inclusive participation and foster equitable partnerships. These barriers were identified as having the potential to negatively affect service delivery [22]. Integrated data systems that are key enablers for data sharing [58] are scarce in Blackpool and the Fylde Coast, which are complicating the generation of positive organizational priorities and subsequently blocking the coordinated efforts of service provision. High levels of deprivation are likely to slow the progress to make improvements, and ICSs may prove to be less effective in their continued involvement, in terms of broad strategic improvements, to address the specific needs of populations [53, 59].

The challenges experienced in relation to integrating care owing to conflicting accountabilities between NHS Boards and local authorities in Scotland highlighted the importance of clear governance and accountability structures [60]. In England, similar tensions have emerged between health and wellbeing boards (HWBs), established in 2013, and the evolving integrated care systems (ICSs) under the Health and Care Act 2022 [61]. HWBs were originally intended to drive local integration, but the creation of ICSs has, in some cases, led to overlapping roles and fragmented accountability. As these systems evolve, there is a clear need to define governance arrangements that clarify responsibilities, strengthen collaboration and ensure alignment across local and regional structures [61]. Also, the Spanish model of Badalona Serveis Assistencials of electronic health records across services demonstrated that integration may serve as a catalyst for overcoming barriers [62]. In Blackpool and the Fylde Coast, PBPs that focus on more specific areas can bridge gaps through redesigning services and foster relationships across organizations, making them more effective to serve community-based needs [63]. The facilitation of such efforts aligns with national frameworks including the NHS Long-Term Plan [7, 8], which advocates for integrated, place-based care models suited for local needs. Consequently, the results demonstrated a low reciprocity rate of 27%, highlighting a lack of mutual recognition of partnerships, potentially affecting the ways resources are shared and whether benefits of coordinated care are actualized [24, 26]. This may be a legacy of the absence of an infrastructure organization. This has meant that VCFSEs are constantly in competition rather than working collaboratively for the benefit of the sector. The improvement of these factors across organizations could prove to be a positive step to address organization-based cultural barriers to enhance community-based collaborations.

### Integration to tackle social determinants of health

The aims of ICSs are to improve health and social care inequality and, consequently, health outcomes by facilitating partnerships [53]. The centralization observed in the findings (meaning that some organizations played a more pivotal role in the network) mirror the challenges of other integrated care models, for example, in Germany and the Netherlands. Germany’s Gesundes Kinzigtal programme focused on aligning financial incentives across healthcare providers to avoid centralization through equitable access to resources, subsequently aiding collaboration [62]. Similar financial and policy alignment strategies might be beneficial in Blackpool and the Fylde Coast to support more equitable share of the resources and potentially aid more collaborations [62]. Counteracting the dominance of a few central service domains was demonstrated via the Dutch U-PROFIT programme, whereby service providers were given more autonomy because of bottom-up collaborative processes [15, 23, 64, 65].

The Core20PLUS5 initiative [66] was introduced to help ICBs take targeted action in addressing health inequalities. Health inequalities, as defined by the National Institute for Health and Care Excellence [67], represent systemic and avoidable disparities in health outcomes among different societal groups. These inequities are not merely byproducts of individual choices but are deeply rooted in socioeconomic structures that influence disease risk, preventive behaviours and access to care. The Dahlgren–Whitehead Rainbow Model (1991) continues to be a key framework for understanding factors contributing to health inequalities [68], highlighting various layers of health influences – including lifestyle, community, living and working conditions and broader social factors – interacting at multiple levels. Tackling the social determinants of health and providing personalized care requires a whole-system approach that fosters better collaboration among various service domains and providers. Consequently, a well-integrated PBP is ideally positioned to address health inequalities and tackle the challenges present locally across social, economic and environmental conditions. We found significant interactions between housing, shelter and nutritional support and mental health, as well as housing, shelter and nutritional support and child and family support, which indicate cross-sector partnership across multiple service domains. This highlights the interaction between multiple factors underpinning complex needs in areas of high deprivation that require a joint response from service providers.

This study confirmed the role of the VCFSE organizations in addressing social determinants of health through providing a broad range of accessible community-based services [69, 70]. The sector’s close ties to the communities and their ability to deliver interventions that focus on the everyday needs [71–73] make them crucial players in integrated care models [74]. However, the findings also revealed a tendency for VCFSE organizations to collaborate primarily within their own service domains, a pattern known as homophily. Homophily can limit an integrated system from addressing broader social determinants of health and lead to fragmented service delivery. In contrast, between-service domain collaborations can strengthen support networks through dividing responsibilities on the basis of strengths [24], sharing best practices and knowledge [75] and amplifying the sector’s voice [30].

Supporting this, the Netherlands’ U-PROFIT programme facilitates organizations closely working together in an integrated care network. This approach improved care for service users whilst supporting collaboration across sectors, ensuring that no single domain operated in isolation [64]. Enhancing the VCFSE sector’s capacity to collaborate effectively across the health and social care network may improve their impact on service access, overall health outcomes and health inequalities [63].

Recent legislative efforts, such as the Health and Care Act 2022 [59], are driving collaborative working within the health and social care service domains. This structural reform aims to generate a more integrated approach to care and improve the alignment of services with the needs of communities through reversing previous policies that emphasized competition [59]. It may just be in line with providing a platform to reintegrate isolated service domains and organizations into relevant service networks. However, translating legislations into practice may prove to be challenging in contexts where systemic disadvantages, austerity and complex health needs are present. It appears that a beneficial approach is strengthening PBPs in relevant areas that involve current and potential key organizations from all service domains. Such partnerships are more likely to be aligned with the community’s needs, and therefore could be more effective compared with region-wide initiatives in addressing community-based difficulties [63]. These efforts are supported by the White Paper on Integration and Innovation [58], particularly in relation to integrating care cross health-based organizations. In Spain, the Badalona Serveis Assistencials was found to improve data sharing practices and coordination across organizations with disjointed connections [62]. Subsequently, strengthening, establishing and renewing partnerships could be supported by better data integration and sharing via the implementation of shared platforms, which would enable organizations to access relevant information, improve monitoring and evaluation and consequently enhance service provision. Furthermore, VCFSE-based capacity-building initiatives and addressing cultural and organizational barriers can help bridge these gaps [22] through training programs, workshops and funding support [75] to ultimately create a more cohesive and resilient care network.

### Methodological strengths and limitations

This is the first study that has used SNA to explore integration and cross-sector domain collaboration patterns across community-based health and social care-based providers in a context of high deprivation in the United Kingdom. Previous SNA research focused on either the structural properties of social networks or their impact within specific domains in relation to collaborations among organizations [39–43]. The current study combined these perspectives and applied SNA to collaborations across diverse service providers. This approach advances the methodological and theoretical application of SNA and provides empirical evidence to inform real-world health and social care strategies relevant to policy and practice. In addition, the current research included a diverse range of organizations, from small representatives of VCFSE organizations to large healthcare providers, which promoted the role and importance of potentially overlooked primary care-based stakeholders [29, 31]. The study addressed the possibility of the bias in the findings through analysing the entire network and the subset of organizations who responded, which allowed for a more nuanced interpretation of the collaborations. This study used multiple sources, including local expertise, to identify community-based services, which enhances the likelihood of generating a comprehensive map of the area.

Additionally, the full list of organizations mapped during this study was shared with participating organizations, which has the potential to raise awareness of existing service provision and enhancing collaborations across organizations. The generated dataset is also available to feed into local authority and ICS directories and therefore has the potential to aid referrals to community-based services and support the work of social prescribing link workers, community navigators and general practitioners (GPs).

The study has several limitations. The data collected relied on self-reported data from a limited number of organizations, 44 out of 453, which may reflect response bias. In addition, participating organizations may have a higher number of collaborations than nonparticipating ones, since collaborations were often mentioned by just one of the parties, even when both organizations participated in the study (that is, reciprocity rate was low) [76]. It is therefore important to first investigate the roots of this low level of reciprocity and, second, the causes of a relatively low participation rate of organizations in the study. Regarding the first issue, it is possible that each person working at an organization is not necessarily aware of all the collaborations the organization has, especially when the organization is larger or less centralized. It may be important to seek responses with the contribution of multiple people within the same organization to get a fuller picture of the diverse set of collaborations an organization may have. Collecting data from organizations as a group rather than through individual surveys may help to reduce the likelihood of reporting bias. Regarding the second issue, this was potentially the result of several factors, including lack of staff time, financial constraints, high workloads, differences in priorities and technical barriers within these organizations. This reflects the broader systemic issues that likely hindered the capacity of organizations to participate in research, subsequently affecting the comprehensiveness of the data collected and the generalizability of the findings [15, 16, 77]. Hence, the primary data collection focused solely on the existence of collaboration, and the results did not provide insights into the quality of these collaborations [47]. To overcome the challenge of low reporting, the study gathered additional data from organizational websites. There is a risk of websites being outdated, which has also been emphasized by Duncan et al. as a potential limitation of the mapping. Indeed, the funding landscape for practitioners in small VCFSE organizations is precarious, and the work is often carried out on a short term, project-by-project basis [78]. Moreover, the uneven impact of austerity on small VCFSE organizations, especially in deprived areas with high demands on services, is well-documented [79]. Therefore, it is possible that the searches identified organizations that no longer operated, which may have contributed to low response rates.

## Future research

Future studies may benefit from examining the evolution of collaborations over time and the factors that contribute to such changes. These explorations may provide further insights into the sustainability of cross-sector and interorganizational networks. Future research on investigating the impact of digital platforms on collaboration patterns could also provide avenues for conceptualization of more effective strategies that promote enhanced community-based care. Moreover, evidence is lacking in relation to factors that facilitate and limit organizational collaborations between health and social care providers. Exploring the role of leadership, organizational cultures and resource allocation, within and across service domains can provide an objective viewpoint to pair with the perspectives of service users and communities to better understand their experiences [47].

## Conclusions

Enhancing the effectiveness of PBPs appears to be the way forward to overcome the barriers that limit the integration of care services. Legislative reforms, including the Health and Care Act 2022 and the White Paper on Integration and Innovation, provide frameworks to aid these efforts. However, their success depends not only on the implementation of organizational processes, sufficient data-sharing practices and capacity-building initiatives but also on ensuring that the voluntary, community, faith and social enterprise (VCFSE) sector has the necessary infrastructure to participate fully in these systems. Without such infrastructure – particularly mechanisms for coordination, collaboration and resource-sharing – the VCFSE sector risks being underutilized, despite its proven capacity to address complex community needs effectively.

For ICSs and PBPs to deliver integrated, resilient care networks, investment in infrastructure that supports the VCFSE sector must become a priority. This would allow for improved collaboration across service domains, better alignment with community priorities and a more effective response to persistent inequalities. Addressing these gaps and barriers can enable policymakers and practitioners to strengthen ongoing efforts, building networks that are both interconnected and responsive to the specific needs of diverse communities.

## Data Availability

The data presented in this study are available upon request from the corresponding author.

## Abbreviations

ICSs: Integrated care systems
ICBs: Integrated care boards
ICPs: Integrated care partnerships
NHS: National Health Service
VCFSE: Voluntary, community, faith and social enterprise sector
LEP: Lancashire enterprise partnership
PACS: Primary and acute care systems
PBPs: Place-based partnerships
TIDieR: Template for intervention description and replication
MRQAP: Multiple regression quadratic assignment procedure

## Footnotes

1 Blackpool Opportunity Area Programme [80], Lancashire Enterprise Partnership (LEP) [81], Blackpool’s Better Start Programme [82], Healthier Lancashire and South Cumbria Integrated Care System [83], Blackpool Council’s Housing Strategy [84], Fylde Coast New Care Model Vanguard [85] and the Health Determinants Research Collaboration [86].
